# The Lethal Toxin from Australian Funnel-Web Spiders Is Encoded by an Intronless Gene

**DOI:** 10.1371/journal.pone.0043699

**Published:** 2012-08-22

**Authors:** Sandy Steffany Pineda, David Wilson, John S. Mattick, Glenn F. King

**Affiliations:** Institute for Molecular Bioscience, The University of Queensland, St Lucia, Queensland, Australia; Universidad Nacional Autonoma de Mexico, Instituto de Biotecnologia, Mexico

## Abstract

Australian funnel-web spiders are generally considered the most dangerous spiders in the world, with envenomations from the Sydney funnel-web spider *Atrax robustus* resulting in at least 14 human fatalities prior to the introduction of an effective anti-venom in 1980. The clinical envenomation syndrome resulting from bites by Australian funnel-web spiders is due to a single 42-residue peptide known as δ-hexatoxin. This peptide delays the inactivation of voltage-gated sodium channels, which results in spontaneous repetitive firing and prolongation of action potentials, thereby causing massive neurotransmitter release from both somatic and autonomic nerve endings. Here we show that δ-hexatoxin from the Australian funnel-web spider *Hadronyche versuta* is produced from an intronless gene that encodes a prepropeptide that is post-translationally processed to yield the mature toxin. A limited sampling of genes encoding unrelated venom peptides from this spider indicated that they are all intronless. Thus, in distinct contrast to cone snails and scorpions, whose toxin genes contain introns, spiders may have developed a quite different genetic strategy for evolving their venom peptidome.

## Introduction

Spiders are one of the most megadiverse animal groups on the planet. They are by far the most speciose venomous animal, with 3,859 genera comprising 42,751 species having been described to date [1]. Their evolutionary success is due in large part to the evolution of a pharmacologically complex venom that enables rapid subjugation of prey and predators. Spider venoms are complex chemical cocktails composed of salts, low molecular weight inorganic compounds (such as polyamines), disulfide-rich peptides (2–9 kDa), and proteins (including enzymes) larger than 10 kDa [2]–[7]. However, most spider venoms are dominated by disulfide-rich peptide neurotoxins that typically have very high affinity and selectivity for specific types of ion channels and receptors, which has made them particularly valuable as pharmacological tools and therapeutic leads [8]–[11].

**Figure 1:**
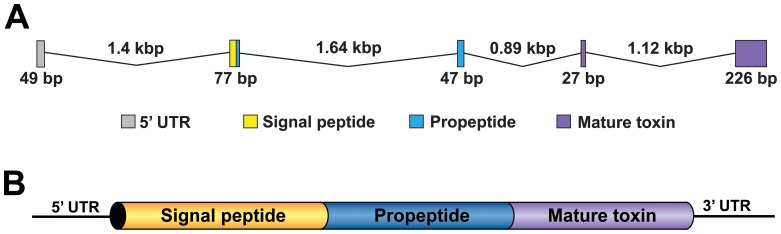
Architecture of spider-toxin genes. Intron-exon organization for genes encoding (A) μ-diguetoxin-Dc1a from the American desert spider *Diguetia canities* [15]; (B) 24 different disulfide-rich venom peptides from the Chinese tarantulas *Haplopelma hainanum* and *Haplopelma huwenum* [16]–[18]. In panel (A), the colors denote exons encoding the signal peptide, propeptide, and mature toxin. In panel (B), the entire toxin prepropeptide precursor is encoded by an intronless ORF.

Spider venoms are predicted to contain more than 10 million bioactive peptides based on their extraordinary taxonomic diversity and the demonstration that some venoms contain >500 unique peptides [12], [13]. Indeed, their venoms can be viewed as pre-optimized combinatorial peptide libraries that have evolved over hundreds of millions of years [14]. Surprisingly, very little information is available about the genetic framework underpinning production of these complex peptide libraries. Numerous transcriptomic studies have revealed that spider-venom peptides are typically produced from a prepropeptide precursor that is posttranslationally processed to yield the mature toxin [14]. However, the sequences of genes encoding spider-venom peptides are available from only the araneomorph spider *Diguetia canities* and the mygalomorph spiders *Haplopelma huwenum and H. hainanum* [15]–[18] (Figure 1).

The first spider-venom peptide gene to be sequenced was that encoding μ-diguetoxin-Dc1a (DTx 9.2) from *D. canities* [15]. The intron-exon arrangement of this gene is similar to that reported for genes encoding peptide toxins in cone snail venoms [19] with small exons (27–226 bp) encoding the signal, propeptide, and mature toxins regions separated by much larger introns (0.89–1.64 kbp) (Fig. 1A). In contrast with cone snails, however, the propeptide and mature toxin sequences are not encoded on single exons but rather are partitioned over two exons separated by a large intron. Nevertheless, this early work suggested that cone snail and spiders may have developed similar genetic strategies for evolving their panel of venom peptides. However, recent work on the mygalomorph spiders *H. huwenum* and *H. hainanum* has shown that, in contrast with cone snails, the genetic architecture of genes encoding venom peptides in spiders is much more variable since all of the toxin genes sequenced from these spiders are intronless (Fig. 1B) [16]–[18].

**Figure 2:**
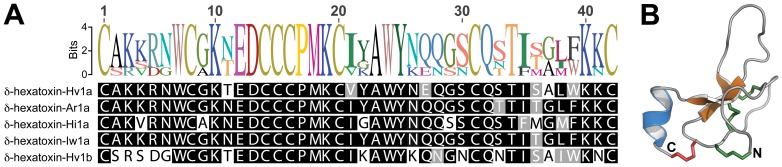
Primary and tertiary structure of δ-hexatoxins. (A) Alignment of the amino acid sequences of the lethal toxins δ-hexatoxin-Hv1a (and its paralog δ-hexatoxin-Hv1b), δ-hexatoxin-Ar1a, δ-hexatoxin-Hi1a, and δ-hexatoxin-Iw1a from the Australian funnel-web spiders *Hadronyche versuta*, *Atrax robustus*, *Hadronyche infensa*, and *Illawarra wisharti*, respectively. Identical amino acids are boxed in black and conservative substitutions are shaded grey. (B) Ribbon representation of the three-dimensional structure of δ-hexatoxin-Hv1a (PDB code 1VTX) [26]. β-Strands and 3_10_-helix are shown in orange and blue, respectively. The N- and C-termini are labeled. The three disulfide bonds shown in green form an inhibitor cystine knot motif while the disulfide bridge that connects the C-terminal Cys residue to the core ICK region is colored red.

Despite their fearsome reputation, only six genera of spiders are capable of inflicting lethal envenomations in humans [20]. The Australian funnel-web spiders (Aranae: Mygalomorphae: Hexathelidae: Atracinae) are a group of approximately 40 species that comprise three of these lethal genera: *Atrax*, *Hadronyche*, and *Illawarra* [21], [22]. Representatives from the genus *Atrax* caused at least 14 human deaths between 1927 and the introduction of anti-venom in 1980 [23]. The envenomation syndrome caused by bites from these spiders is due to a 42-residue peptide known as δ-hexatoxin (formerly δ-atracotoxin) (Fig. 2A). This peptide induces delayed inactivation of voltage-gated sodium channels, resulting in prolonged action potentials that cause massive neurotransmitter release from both somatic and autonomic nerves [24], [25]. The structures of δ-hexatoxin-Hv1a and δ-hexatoxin-Ar1 (from *Hadronyche versuta* and *Atrax robustus* respectively) were solved using NMR spectroscopy [26], [27]. Structurally, the toxins are stabilized by four disulfide bonds, three of which form an inhibitor cystine knot (ICK) motif [28], [29]; the fourth disulfide bridge links the C-terminal residue to the core ICK region of the peptide (Fig. 2B).

Unfortunately, no information is currently available about the genes encoding δ-hexatoxin or any other neurotoxins produced by any species of Australian funnel-web spiders. To investigate the architecture of the gene encoding δ-hexatoxin, we cloned and sequenced its genomic DNA from the Fraser Island funnel-web spider *Hadronyche infensa*, as well as the genes encoding two other neurotoxins produced by the same spider.

## Materials and Methods

### Spider collection

Adult and juvenile specimens of the Australian funnel-web spider *Hadronyche infensa* (Hickman) (Aranaea: Hexathelidae) were collected from Orchid Beach, Fraser Island, Queensland, Australia. The specimens were collected from private land with the permission of the owners (the Tasker family) and were not endangered species or subject to any specific collection permits. Individual spiders were housed in plastic containers at 23°C in dark cabinets until required. Throughout this manuscript, spider taxonomy is from the World Spider Catalog V. 11.5 [1] and toxins are named following the rational nomenclature recently proposed for spider-venom peptides [30].

**Figure 3:**
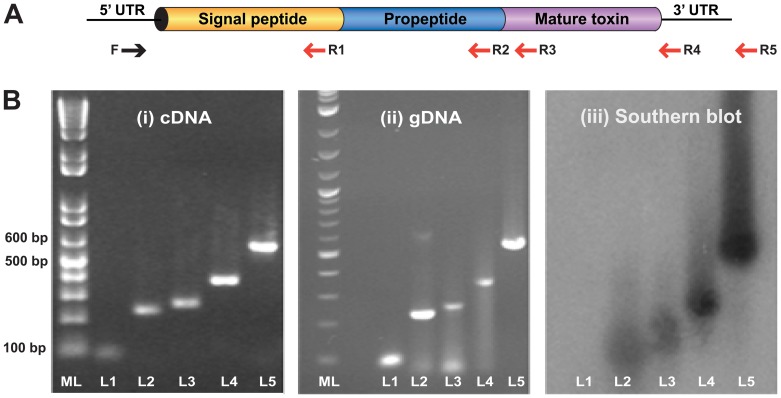
The gene encoding δ-hexatoxin-Hi1a is intronless. (A) Schematic of the putative δ-hexatoxin gene showing the region that each primer set (L1–L5) is designed to amplify. (B) Gels showing the PCR products obtained using each of the designed primer sets L1–L5: (i) cDNA template; (ii) gDNA template; (iii) Southern Blot. For each gel, ML denotes 1 kb molecular-weight ladder, while L1–L5 denote the primer sets A–E shown in Table 1.

**Table 1:** Primer sets used to amplify specific regions of the transcript and gene encoding δ-hexatoxin-Hi1a from cDNA and gDNA templates, respectively (letters in brackets denote lanes in gels in Fig. 3B).

Primer set	Target region	Predicted size (bp)
**A (L1)**	Signal to the beginning of the propeptide sequence	90
**B (L2)**	Signal to the end of the propeptide sequence	219
**C (L3)**	Signal to the beginning of the mature sequence	243
**D (L4)**	Signal to the end of the mature sequence	345
**E (L5)**	Signal to the end of the 3′ untranslated region	560

### Isolation of genomic DNA

Genomic DNA (gDNA) was extracted from the leg muscle of a number of *H. infensa* specimens using the method described previously [31]. Briefly, three legs were isolated from the base of the cephalothorax and ground with a chilled, sterile mortar and pestle in liquid nitrogen. After pulverization, the tissue (∼100 mg) was added to 400 μl of low salt buffer (0.4 M NaCl, 10 mM Tris-HCl pH, 2 mM EDTA pH 8, 2% SDS and 400 μg/ml proteinase K) and incubated at 55°C overnight. DNA was recovered by adding 300 μl of 6 M NaCl followed by ethanol precipitation. DNA was re-suspended in 250 μl of DNAse/RNAse-free water. The quality and quantity of gDNA was determined visually using a 1% agarose gel stained with SYBR® Safe and by assessing the A260/280 ratio using a spectrophotometer. Isolated gDNA was used as the template for PCR.

### Preparation of venom-gland cDNA

Venom glands from one *Hadronyche infensa* spider were removed from an anesthetized specimen and placed immediately in TRIzol® reagent. Total RNA was extracted according to the manufacturer’s protocol. The quality and quantity of RNA was determined by running an aliquot on a Pico chip in a Bioanalyzer (Agilent) and by assessing the A_260_/A_280_ ratio. mRNA was reverse transcribed using SuperScript® III reverse transcriptase (Invitrogen) using the following two-step reaction: (a) 1 μl of total RNA was mixed with 2.3 μM *Not*I-(dT)_18_ primer, 1x dNTPs, and nuclease-free water to a volume of 13 μl, then incubated at 65°C for 15 min followed by 1 min on ice; (b) The following reagents were then added: 4 μl of 5× first strand buffer (250 mM Tris-HCl pH 8.3 at room temperature, 375 mM KCl and 15 mM MgCl_2_), 0.005 M dithiothreitol (DTT), 1 U SuperScript® III and nuclease-free water to a volume of 20 μl. The sample was then incubated as follows: 37°C for 10 min, 42°C for 10 min, 50°C for 30 min, 55°C for 15 min, and a final cycle of 75°C for 15 min. Nuclease-free water was then added to a final volume of 50 μl. The resulting cDNA was used as a template for PCR.

**Figure 4:**
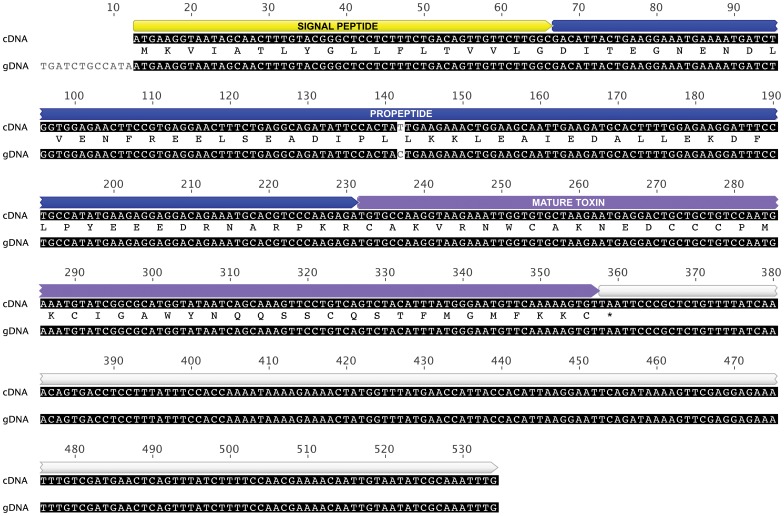
Architecture of gene encoding the lethal toxin from Australian funnel-web spiders. Alignment of the cDNA and gDNA sequences obtained for δ-hexatoxin-Hi1a. The nucleotide sequences are identical except at a single position (T versus C, highlighted in grey) that does not alter the encoded protein sequence. The stop codon is denoted by an asterisk. A schematic of the toxin precursor showing the signal peptide, propeptide, mature toxin, and 3’ untranslated region in yellow, blue, purple and white, respectively, is shown above the sequences. The protein sequence (i.e., a translation of the cDNA/gDNA) is shown sandwiched between the cDNA and gDNA sequences.

### Amplification of the δ-hexatoxin gene and gene transcript

PCR reactions using gDNA as template were carried out using a long-distance amplification polymerase (NE biolabs). Each 50 μL PCR reaction contained 100 ng of gDNA template, 1× PCR buffer, 0.2 μM each dNTPs, 0.5 μM forward primer, 0.66 μM reverse primer and 1 U *LongAmp* polymerase. Cycling conditions were as follows: 2 min at 95°C followed by 40 cycles of 95°C for 30 s, 50°C for 1 min, 65°C for 2 min, and a final cycle of 65°C for 10 min.

**Figure 5:**
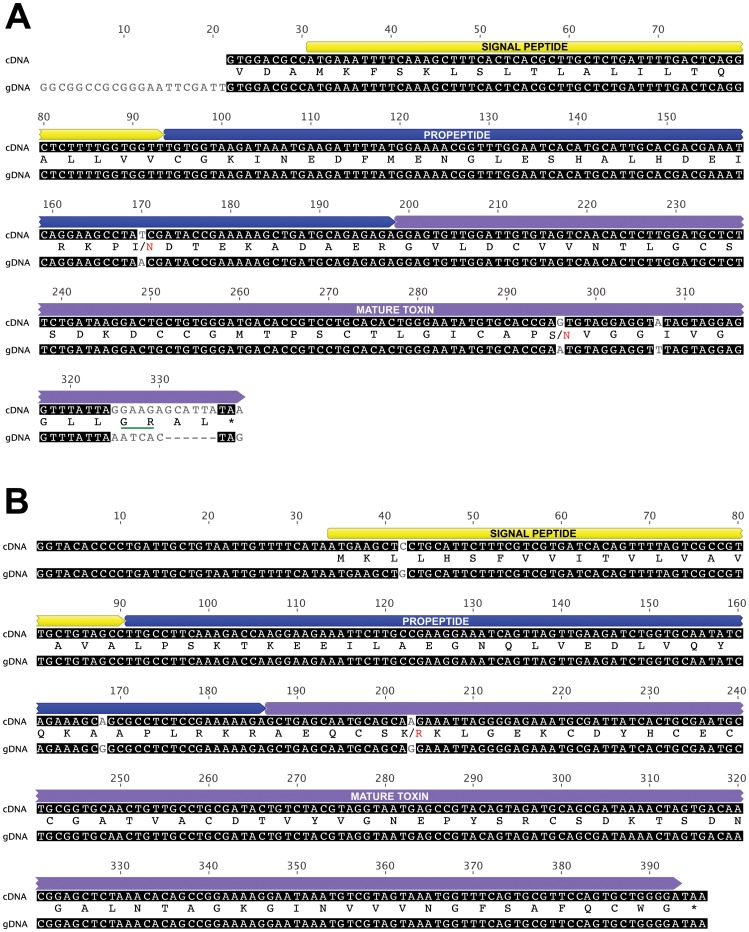
Architecture of genes encoding ICK toxins from Australian funnel-web spiders. Alignment of cDNA and gDNA sequences of (A) ω-hexatoxin-Hi2a and (B) U_3_-hexatoxin-Hi1a. Stop codons are indicated by an asterisk. A schematic of the toxin precursors showing the signal peptide, propeptide, and mature toxin in yellow, blue, and purple, respectively, is shown above the sequences. The protein sequences (i.e., a translation of the cDNA/gDNA) are shown sandwiched between the cDNA and gDNA sequences. There are several codons where a difference between the cDNA and gDNA sequences leads to a difference in the sequence of the encoded protein; in these cases the amino acid encoded by the cDNA and gDNA are shown in black and red, respectively. The “GR” sequence underlined in green at the C-terminus of the mature-toxin region of ω-hexatoxin-Hi2a in panel (A) is a C-terminal amidation signal [34].

Platinum® *Taq* DNA polymerase (*Taq* Pol) (Invitrogen) was used for PCR using cDNA as template. Each 50 μL reaction sample contained 100 ng of template, 1× PCR buffer, 0.2 μM each dNTPs, 0.5 μM forward primer, 0.66 μM reverse primer, 1 U *Taq* Pol and 1 mM MgCl_2_. Cycling conditions were: 2 min at 96°C followed by 35 cycles of 94°C for 45 s, 50°C for 1 min, 72°C for 2 min, and a final cycle of 72°C for 10 min.

For both sets of PCRs, 10 μl of PCR product was run on a 1.5% agarose gel stained with SYBR® Safe and visualized using a BioRad Gel Doc™ system. PCR products encoding full-length δ-hexatoxin were purified using a QIAquick PCR purification kit. Purified samples were sent for sequencing to the Australian Equine Genetics Research Centre at the University of Queensland. Chromatograms were analyzed using Geneious Pro V. 5.4.5. cDNA and gDNA sequences were aligned using ClustalW [32].

### Southern Blot analysis

After amplification, PCR products were concentrated to 10 μl, loaded onto a 1.5% agarose gel and electrophoresed for 1.5 h at 100 V. Gels were photographed, marked and prepared for Southern blotting as described previously [33]. Subsequently, cDNA probes were prepared using the Amersham MegaPrime™DNA labeling system according to the manufacturer’s protocol. Hybridization between the membrane and the ^32^P-labelled cDNA probes was performed overnight at 65°C with hybridization solution (0.263 M Na_2_HPO_4_ pH 7, 1 mM EDTA, 7% SDS, 1% BSA and deionized water). After incubation, membranes were washed two times with a 2× saline-sodium citrate (SSC)/1% SDS solution (10 min each), 15 min with a SSC/1% SDS solution, and two final washes with a 0.2× SSC/1% SDS solution for 15 min each. Radiolabelled membranes were wrapped in a plastic cover, loaded onto a radiography cassette, and incubated at room temperature for 2 h. Screens were scanned using a Typhoon 8600 variable mode imager (GE Healthcare, UK).

## Results

### The gene encoding δ-hexatoxin is intronless

In order to investigate the architecture of the gene encoding the lethal δ-hexatoxin from the Australian funnel-web spider *Hadronyche infensa*, the sequence of a previously isolated cDNA transcript encoding a δ-hexatoxin precursor was obtained from the ArachnoServer database (www.arachnoserver.org) [13] and used to design primer sets as shown in Table 1 and depicted in Fig. 3A. Each primer set was designed to target and amplify a specific segment of the cDNA precursor encoding δ-hexatoxin with the aim of amplifying its gDNA counterpart in such a manner that if the gene contained introns one would observe differences in size between the amplified gDNA and cDNA segments. The same forward primer was used for each amplification, whereas the reverse primer was varied in order to obtain stretches of sequence corresponding to either: (i) the signal peptide; (ii) from the beginning of the signal sequence to the end of the propeptide; or (iii) from the beginning of the signal peptide to the end of the mature toxin.

Utilizing this targeted amplification approach, different regions of the cDNA precursor encoding δ-hexatoxin-Hi1a were amplified as shown in Fig. 3A; amplifications were repeated using the same primer sets but with the PCR template changed to gDNA instead of cDNA. Comparison of the amplified cDNA fragments (Fig. 3A, panel i) with those amplified from the gDNA template (Fig. 3B, panel ii) revealed that the fragment sizes are identical for each primer pair, suggesting that the gene encoding δ-hexatoxin-Hi1a is intronless.

Due to the manner in which spider-venom genes have been recruited to the venom arsenal and massively amplified and diversified, spider venoms typically numerous paralogs of each toxin [14]. Thus, it is possible that the PCR products described above might have resulted from non-specific primer binding to closely related targets rather than the δ-hexatoxin-Hi1a gene itself. With the aim of investigating whether all of the amplified products correspond to different sections of the precursor within their respective templates (gDNA or cDNA), southern blot hybridizations were performed. If a radiolabelled probe, in this case the cDNA, hybridizes with the immobilized template, the complementarity would be indicative of true amplifications of the desired target. As shown in Fig. 3B, panel iii, the full-length gene encoding δ-hexatoxin-Hi1a hybridizes with the labelled cDNA probe, proving that the amplified gene corresponds to that of the lethal δ-hexatoxin-Hi1a. Furthermore, as shown in Fig. 4, we found that the gDNA sequence was identical to its cDNA counterpart except for a single T
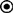
C base substitution that does not alter the encoded protein sequence.

### Genes encoding non-lethal Australian funnel-web spider toxins are also intronless

Using a simplified version of the reaction scheme above, single reactions were performed with one forward and one reverse primer to amplify two other toxin genes encoding non-lethal peptides from the venom of *Hadronyche infensa*, known as ω-hexatoxin-Hi2a [34] and U_3_-hexatoxin-Hi1a. Each toxin gene was amplified, cloned and sequenced independently. As shown in Figures 5A and 5B, both of the genes are intronless. The cDNA and gDNA sequences encoding ω-hexatoxin-Hi2a were identical except for a single nucleotide difference in each of the propeptide and mature-toxin regions, and an apparent deletion of two codons at the C-terminus of the mature-toxin (Fig. 5A). However, this two-codon deletion is immediately preceded by a “GR” C-terminal amidation signal [34], so it would have no impact on the size of the mature toxin. In the case of U_3_-hexatoxin-Hi1a, the cDNA and gDNA sequences are identical except for a single A?G base substitution that corresponds to a conservative amino acid change from K to R at position 6 in the mature toxin (Fig. 5B).

## Discussion

The venoms of many animals, including marine cone snails, scorpions, snakes, sea anemones, spiders and platypus contain many peptides that are produced by post-translational processing of a larger precursor [14]. The precursors of cone snail and sea anemone toxins contain both a signal sequence and a propeptide region, whereas the propeptide region is generally absent in scorpion and snake toxin precursors [35], [36]. The situation is more complex in spiders, with propeptide regions typically present in transcripts encoding “short” toxins (<5 kDa) [4], [17], [34], [37], [38] but absent from transcripts encoding longer toxins [39]. However, in all cases, the precursor encodes only a single copy of the mature toxin sequence.

Since most venom research (not only on spiders) has been focused on the isolation and identification of peptides via proteomic and transcriptomic approaches, mainly with a view towards developing new pharmacological tools and therapeutic leads, there is a paucity of information on the number of toxin genes, their genetic architecture, and the genetic mechanisms underpinning toxin diversification. A small number of genes encoding venom peptides have been described from cone snails, scorpions, sea anemones and snakes and with only a few exceptions they all have an intro-exon architecture. In conotoxins, one intron is positioned between the signal and propeptide coding regions and another between the propeptide and mature toxin region [19], whereas a variable number of introns (1 to 3) can be found in snake-toxin genes [35], [40], [41]. There are examples of both intronless and intron-containing venom genes in scorpions [36], [42]–[44].

The gene encoding μ-diguetoxin-Dc1a from the araneomorph spider *D. canities* [15] was the first spider-venom peptide gene to be sequenced. This gene contains five introns that span 5.5 kb; the first two introns are located within the 5′ UTR and propeptide-encoding region while the other two are situated in the region encoding the mature toxin (Fig. 1A). More recently, 24 genes encoding ICK-containing venom peptides were sequenced from two mygalomorph spiders, namely the Chinese bird spider *Haplopelma huwenum* and the Chinese Black Earth Tiger tarantula *Haplopelma hainanum* [16]–[18]. All of these genes lacked introns, in striking contrast to the gene encoding μ-diguetoxin-Dc1a.

In this study we determined for the first time the architecture of genes encoding ICK-containing venom peptides from the highly venomous Australian funnel-web spider. All three genes we examined were intronless, including the gene encoding the lethal δ-hexatoxin-Hi1a peptide. Thus, to date, all of the 27 genes encoding ICK-containing venom-peptides that have been sequenced from primitive mygalomorph spiders are intronless, which raises interesting questions about the ancestral state of genes encoding spider-venom peptides as well as the mechanism of toxin diversification.

Spiders evolved from an arachnid ancestor more than 300 million years ago (Mya). Extant spiders are divided into the suborders Opisthothelae and Mesothelae, with the latter comprising a single family of primitive, venom-less burrowing spiders [45]. Opisthothelae is further divided into two infraorders, Mygalomorphae and Araneomorphae, sometimes referred to as “primitive” and “modern” spiders, respectively [45]. Molecular clock analyses suggest that mygalomorphs and araneomorphs split ∼280 Mya [46], which is consistent with the fossil record. The earliest mygalomorph fossil dates to the early Triassic period ∼240 Mya, while the earliest araneomorph fossils date to ∼225 Mya [47]. If the complex intron-exon architecture determined for μ-diguetoxin-Dc1a is representative of genes encoding araneomorph toxins then either introns were lost secondarily by mygalomorph spiders following their divergence from araneomorphs more than 250 Mya or the ancestral state of spider-venom peptide genes is intronless.

Minimization of genome size through extensive loss of ancestral introns has been reported for microsporidia, fungi, red algae, and apicomplexans [48]. At this stage it is unclear whether there has been tendency towards minimization of genome size in mygalomorphs, but this seems unlikely. Recent surveys of ∼120 araneomorph spiders [49], [50] have revealed an enormous variation in genome size (700–5,500 Mb) but there are no current estimates for genome size in mygalomorph spiders. Loss of introns is associated with evolutionary diversification of snake-venom disintegrins but in this case intron loss is correlated with a corresponding reduction in protein size [51]. Given the growing consensus that the ancestral bilaterian was rich in introns and that differences in intron numbers between animals largely reflect different levels of intron loss [48], the most parsimonious explanation of the current sparse data on spider-venom peptide genes is that ancestral spider-toxin genes contained introns (as seen in the gene encoding μ-diguetoxin-Dc1a) but these were lost at an early stage in the evolution of genes encoding ICK toxins. Since Australian funnel-web spiders (family Hexathelidae) and tarantulas (family Theraphosidae) diverged more than 200 Mya, mygalomorph spiders presumably dispensed with introns in genes encoding ICK toxins at a very early stage of venom evolution.

The implications of intron loss or gain in genes expressing spider-venom peptides will not be understood until more genes are sequenced from a greater diversity of spiders. However, the apparent widespread loss of introns in genes encoding mygalomorph ICK toxins raises questions about the mechanism of toxin diversification, as alternative splicing and other intron editing mechanisms are clearly not being used to expand the repertoire of venom peptides. Moreover, the absence of introns makes it difficult to invoke exon shuffling as the mechanism by which spiders created larger “double-knot toxins” comprised of two tandemly-repeated ICK domains [52].

The absence of introns suggests that the mechanism of diversification of spider-venom ICK toxins differs from that employed by venomous cone snails to expand their toxin repertoire. As for spider-venom ICK toxins, disulfide-rich peptides from cone snail venoms are initially produced as prepropeptides that are post-translationally processed to yield the mature toxin. These cone snail toxins are encoded by genes that architecturally resemble those encoding the spider-venom peptide μ-diguetoxin-Dc1a, with three exons separated by large (>1 kb) introns [19]. Exon III, which encodes the mature toxin, appears to have evolved at a 10-fold higher rate than exon I, which encodes the signal peptide, and it has been suggested that the separation of these exons by much larger intronic sequences has facilitated their markedly different rates of mutation [19]. Spider-venom ICK toxins show a similar disparity in the rate of mutation between the signal peptide and mature toxin [14], without the benefit of these regions being encoded by separate, widely separated exons. Hence, it will be interesting to determine what allows the mature-toxin region to be extensively mutated over evolutionary time while the signal peptide that is only ∼40–60 bp upstream remains under strong negative selection pressure [14].

Most toxin genes are transcribed at high frequency during venom regeneration, and thus intronless genes might diversify over time due to elevated rates of mutation that are sometime associated with highly transcribed genes, a process known as transcription-associated mutation (TAM). TAM is associated with an increased frequency of mutations such as base replacements, deletions, and recombination [53]. Other heavily transcribed intronless genes such as those involved in immune recognition and response diversify via mechanisms of recombination, somatic hypermutation, class switch recombination, and gene conversion [54], [55]. Whether any of these processes underlie the diversification of intronless spider-venom genes remains to be seen.

In summary, we have shown that the gene encoding the lethal δ-hexatoxin-Hi1a peptide as well as the genes encoding two other ICK-containing toxins (ω-hexatoxin-Hi2a and U_3_-hexatoxin-Hi1a) from the Australian funnel-web spider *Hadronyche infensa* are intronless. This rules out alternative splicing as a mechanism for enhancing venom diversity and it raises still to be answered questions about the ancestral state of spider toxin genes.
